# Indents

**Published:** 1920-10

**Authors:** 


### INDENTS

**☞Brief Articles of Technical or Scientific Value will receive notice and comment. Contributions invited.**

---

DENTAL HYGIENE. In 1894, I made the statement that the time might come when we could use vaccination for the prevention of dental carries just as we were vaccinating for the prevention of smallpox. I was held up to some ridicule at the time, but I felt much better when Dr. Black took me to one side and said: ''My boy, you are not so much astray after all; that time may come.''

We are not much nearer to that consummation than we were in those days. Even if we were, we would not prevent dental diseases at once. Suppose tomorrow a serum should be invented that would actually stop dental decay, it would take a long time to get the profession generally and the public conversant with it, and it would take a longer time to bring it into practical use. And to-day we can not count much on anything of that kind in order to meet the situation as it confronts us. The most tangible thing we have to work upon now is the subject of oral prophylaxis. It is the significant question to-day—the question of oral hygiene.

In this movement it seems to me the greatest thing that we have to think about is not so much the filling of teeth of these poor children and of curing disease as it is their education toward those means by which these diseases may be prevented. We can not hope by our own intervention to prevent these diseases, but we can educate the people so that they can prevent them, and we can accomplish definite results as has been demonstrated in various communities.

Last May I visited Boston and the Forsyth Dental Infirmary. I was very much impressed with what I saw there. I want to pay a tribute to the Forsyth Dental

Infirmary and to the Rochester Infirmary, and particularly to Mr. Eastman and to the Forsyths. In the Forsyth Infirmary I was greatly impressed with the wonderful equipment and the splendid care taken of the children, looking at all times to their comfort, and there was one thing which seemed to me more significant than any other and it was this: When I went to a little room down stairs there were two young ladies who had a line of children standing up and were putting them through a toothbrush drill, and then taking these children one by one and making an inspection, and a report, and tabulating that report to see what attention had been given to the previous instruction. It was very gratifying to see one little child walk up and receive a badge of some kind; it was a recognition of the fact that the child had listened to the instruction and had cared for her teeth. It is the education of the children so that they may, by their own efforts, accomplish this matter of prevention.

I am inclined to think in smaller communities greater advances may be made along these lines than we are able to do in these large communities. I have had some experience, and some of my friends have had more experience in the meeting of this situation in our own city. I think probably Dr. J. P. Smith can tell you something about that, but I am going to contrast what the Health Commissioner of the City of Chicago said this morning with something that has happened in another community I am familiar with.

Away down in the South Seas there are two islands which are called New Zealand, in which there are about a million people, whereas, here in the City of Chicago we have more than two million. In New Zealand the state takes charge of the teeth of the children up to a certain age. Our friend, Colonel Rishworth, of Auckland, New Zealand, was passing through from Europe to his home a year ago, and he stated that his plan was to go before Parliament and request an appropriation to this end, that the state should take care of the teeth of the children up to a certain age, regardless of the financial standing of the parents. He said that the state should hire dentists and hold them responsible for the condition of the teeth of the children.

I received a letter from him a short time ago in which he tells me that has been accomplished, and the state is going to see that the teeth of the children of New Zealand are kept in good condition until such time as they have learned how to care for them and to go to their own dentists. This is the most significant thing that has happened in connection with the movement of oral hygiene. It was suggested to Colonel Rishworth that they might appropriate $200,000 a year. He said to me: "I am not going to listen to that; that won't be enough; the smallest appropriation should be half a million dollars," and he writes me that the work is now under way.

We listened a few moments ago to the Health Commissioner of the City of Chicago, which has over two million and a half of people. The City Council he told us, is in session to-day and it has agreed to appropriate $5,000 for dental service. I was never so ashamed of the City of Chicago as I am at this moment. If it had not been for the generosity of the private citizens of Chicago, dental hygiene would not have been introduced in the public schools at all.—Dr. C. N. Johnson, in *Journal N. D. A.*

---

The British Dental Association. The annual meeting at Bournemouth proved to be a pleasant and interesting gathering. Mr. A. E. Ball, the newly-elected President, had held the office of President-elect for no less than six years, as the War, which upset the whole world, also upset the B. D. A. programme. This lengthy novitiate to executive office will not be lost, however, and we look forward to a year of activity under his guidance. The subjects touched upon in his address included a strongly-worded plea for more systematized treatment of the teeth of large sections of the people, the position of voluntary hospitals which were becoming more and more in need of assistance from the state or municipalities, and a hope that when the long-promised bill for the amendment of the Dentists Act was brought forward, it would be discussed in a broad and not a selfish spirit.

The retiring President, Mr. Montagu F. Hopson, who has done splendid work for the Association for many years past, in his valedictory address, drew attention to the fact that the Ministry of Health regards our specialty as an integral part of any scheme of National Health Service. In any such scheme he was sure the profession would play their part in a national and not in a narrow, syndicalist spirit. He hoped that in the coming year the political ferment might be settled and the Association be able to resume its better object, the advancement of the science and practice of dental surgery.

The Council of the League of Nations have approved of the plan submitted for the constitution of an International Health Office, through which is to be secured the organized co-operation of at least forty states of the world in the interests of health. There is every probability that the scheme will be adopted by the Assembly of the League next November, and its non-political and beneficent objects will surely be an alluring and effective invitation to those nations who at present stand outside the League.

The International Health Office will consist of a general committee, delegates appointed by members of the League, together with delegates appointed by countries not members of the League, but represented on the committee of the Bureau of International Public Health. This committee meets at least once a year. There will also be a permanent committee, consisting of delegates of members of the Council, four members elected by the general committee, the president of the general committee, a representative of the League of Red Cross Societies, and a representative of the Governing Body of the International Labor Office.—*The Dental Record.*

---

MAKING ARTIFICAL STONE MODELS. Dry the impression and give it a good coat of shellac varnish and let it dry for an hour, then oil it with any good oil. Wash with cold water and let it stand fifteen minutes. Mix the stone compound as thick as putty and pack it into the impression. Do not move it around as this will make it porous.—L. Balter, *Cosmos,* September.

---

PARTIAL DENTURES. A method which I have found useful in making and inserting partial dentures which are devised to fill undercut spaces left by extraction of one or more teeth is as follows: Flask the denture as usual and pack with the ordinary rubbers till the undercut portion is reached; then fill this in with Ash's soft red or Vela rubber. When the denture is fitted it will be noticed that there is a soft elastic pad that is easily manipulated into the undercut, leaving the denture firmly situated, and not, as I have frequently found when not using this method, that the vulcanized rubber must be cut away to allow the denture to be placed, thus producing an ill-fitting case.—David Baghel, L.D.S., B.D.Sc., in *Commonwealth Dental Review.*

---

THE COMMERCIAL *vs.* SURGICAL AND REMEDIAL APPLIANCES. All articles manufactured in Canada are subject to a levy of one per cent., and Inland Revenue officers in Ottawa have now decided that dental appliances for use in the mouth are subject to this tax. Dental laboratories are informed that they must pay to the Government of Canada one per cent. upon the net invoice price of all such articles made by them. It is further reported that in some instances Revenue officers have "suggested" that the tax applies to prosthetic restorations made entirely by the dentist in his own premises. It will be interesting to learn what attitude dentists in Canada take up in resistance to this novel and most objectionable impost—if indeed it is anything more than the threat of a grabbing tax-gatherer or a too-zealous subordinate official. A whole host of irritating and obstructive fine distinctions and subtleties would arise were the dentist, in the matter of oral restorations and appliances, to be ruled or instructed by Revenue officials as to the precise point at which purely professional service ended and the process of fabrication began. Many of the materials used have probably already had the tax paid on them at one or more stages of manufacture, and before they became what Revenue officials would call the "raw material" of prosthetic appliances. But this in itself would not suffice to bring dentures or bridges into a unique class for exemption from further taxation. As pointed out here a good many years ago, the whole issue with its important and probably far-reaching consequences turns upon whether a denture (bridge, or the like) can be decided as coming strictly within the class of *manufactured* articles and portable utilities. If dentists as a body have no sincere conviction that there are inherent differences and an unalienable distinction between a denture and a pair of spectacles or an artificial limb, then the whole case is given away.—*The Dental Record.*

---

EXTRACTING UPPER CANINES. Sometimes these are very difficult to extract. When removing the upper teeth for a plate, remove the teeth on either side of the canine and grasp the latter at the sides—the forceps will not slip and the tooth is easily removed.—*Oral Health.*

---

EXTENSION FOR PREVENTION. The outline form comprehends the doctrine of extension for prevention and the esthetic form.

Extension for prevention is that form that is given to the outline of a cavity that will bring the margins of the finished filling into the areas of relative immunity to decay.

We know that there are certain areas of the tooth surface that are not as susceptible to the beginnings of decay as are other parts, and it is the part of wisdom and good operative ability to so arrange our cavity that the finished filling will cause the tooth to present less vulnerable points for the ravages of decay than it did at the beginning of the operation, for if we do not change the condition that called the first decay into being, what assurance have we that there will not be a recurrence of decay sooner or later?

In making our outline form, then, to conform with the doctrine of extension for prevention, we must cut our cavity buccally and lingually so far that the margin will be far enough out of the embrasures that the margin of the finished filling will be perfectly clear of approximating tooth or of filling in the tooth. It must be so far out that it will be self-cleansing; that is, that the bolus of food as it travels down the tooth as it is crushed in the act of mastication, will scour the margin of the filling from occlusal to gingival. It is at the gingival angle that the most care should be taken, for here is the point of greatest vulnerability, and here more than anywhere else is there a recurrence of decay. The older operators insist that this is because there is not the perfect adaptation of the filling here as there is at other points, owing to the difficulty of manipulation, and while that is true to some extent, the fact remains that fillings that are so made that the angles at the gingival margin are brought well into territory that is kept clean do not decay in a degree at all comparable to those that are not so made. It is important, then, that the lines of the cavity buccally and lingually should be brought well out of the embrasures from the occlusal margin down to the gingival angle. The gingival margin should be carried well under the free margin of the gum, for we know that the tooth does not decay under healthy gum tissue. It is well to bear in mind the last part of that sentence—under healthy gum tissue—and be sure that we leave the gum in a healthy condition, for a great deal of harm is done by careless or ignorant operators in mutilating the delicate septal tissue either in the making of a cavity or in the polishing of the filling, or what is worse, leaving a rough gingival margin to the filling or leaving a bad overhang which will serve as a continual source of irritation, which will set up an inflammation, and will cause a congested condition of the gum or will cause it to recede, leaving a vulnerable area for a future recurrence of decay.—J. V. Conzett, in *Journal of the National Dental Association.*

---

FUNCTIONAL ARTICULATION. Traumatic occlusion is malocclusion of bridges and partial dentures by which undue stress upon one or more teeth results from the malrelation of the inclined planes. This condition is frequently attributable to faulty fillings or inlays, and in the absence of such fillings or inlays may be traced to uneven wear of the occlusal surfaces of the natural teeth, or to the malposition or malalignment of teeth resulting from disease. So that we have in the last instance a vicious circle. A state of disease producing traumatic occlusion, and a traumatic occlusion preventing a return to a state of health.

Functional occlusion is a term that might be adopted to indicate such contact of the teeth when the jaws are closed that movement of the mandible with the teeth held in tight contact, or any normal functional movement of the mandible, will cause no undue stress upon any tooth or teeth.

It is manifest that to artificially produce such a relation it is needful that the articulator used should, with absolute accuracy, register all the normal, or even habitual movements of the mandible of the individual for whom the artificial substitutes are to be constructed. Can such an articulator be obtained?

The only absolutely reliable articulator which will record all the normal or habitual movements of an individual's mandible, is the jaws of the patient himself. The procedure then is as follows: Pursue the course which will obtain as accurate results as possible in the laboratory. A separate paper could be written on this subject. But at the moment we are discussing final results only.

The bridge should be constructed with removable porcelains, later to be cemented to place. These porcelains should not be cemented in prior to the technique about to be described. The metal parts having been accurately and satisfactorily adjusted, the most distal porcelain is placed in its box and the patient asked to close his mouth, carbon paper having been inserted between the porcelain and the antagonizing natural teeth. The mandible is then moved about in all natural excursions, the carbon registering the faulty spots on the inclined planes. The porcelain is removed and the discolored spots very accurately ground away with the tiniest of carborundum stones, the inclined planes being thus formed in accommodation with the patient's habits. This operation is repeated until with the porcelain in place, and the forefinger of the operator's hand resting against the buccal surfaces of the porcelain and its natural antagonist, the patient holding his jaws rigidly together and then making slight but forceful lateral movement, fails to unduly move either the porcelain or the natural opposing tooth.

This technique is followed with each porcelain separately, and when all are perfected their occlusal surfaces should be properly carved as to grooves and sulci, and then polished. They are then cemented to the bridge, after which, because of the presence of the cement, it may become necessary to repeat the tests and do some final grinding and adjusting of the inclined planes.—Dr. Ottolengui, in *Items of Interest* for March.

---

Robbing the Child. The *Chicago Tribune* the other day had this significant editorial:

"The finance committee has reversed the order of race preservation. The law of life is that youth must be served. The old must die and the young must live. But the thrifty legislators in the city hall seem to have forgotten this unbending law; they take from the children that political jobs may endure.

"The city council has entirely abolished the appropriation for dental examination in the schools and has reduced the appropriation for physicians and nurses. This is done in the face of a report that a large percentage of Chicago school children are victims of undernourishment and malnutrition.

"Dental examinations in the schools are necessary to the public health. So are the visits of school physicians and nurses. There are few things in the city budget that we can not afford to sacrifice for the sake of these. The finance committee had better wake up."

This false economy being practiced in Chicago is all too common in this generation.—*Journal N. D. Assn.*

---

Vulcanizing on Plaster. E. H. Mathias reports experimental work on the behavior of investment plaster in the vulcanizing of rubber dentures. The author has attempted to determine the amount of expansion and contraction which plaster undergoes in the process of vulcanization. He states that increasing the water-plaster ratio reduces the expansion of plaster, but weakens the strength of the model. He finds that the finer ground plasters are the stronger and that the degree of fineness has little or no effect upon expansion. Vulcanite dentures flasked with flasking compound having plaster of paris as a base should be cooled and removed from the flask at once upon completion of vulcanization. Vulcanite flasks should be equipped with a spring tension to allow for expansion of rubber and a large excess of rubber should not be used. The entire article is worthy of study by those who use plaster of paris.—*Journal N. D. A.* for May.

